# Revealing Monogenic Diabetes: Clinical and Genetic Features of Pediatric MODY Cases in Türkiye: Single Center Experience

**DOI:** 10.1155/pedi/4035026

**Published:** 2025-12-01

**Authors:** Aslihan Sanri, Tugba Kontbay Cetin, Emel Gul Acikgoz, Mehmet Burak Mutlu, Ozlem Sezer

**Affiliations:** ^1^Department of Pediatric Genetics, Samsun Training and Research Hospital, Samsun, Türkiye; ^2^Department of Pediatric Endocrinology, Samsun Training and Research Hospital, Samsun, Türkiye; ^3^Detagen Genetic Diseases Evaluation Center, Kayseri, Türkiye; ^4^Department of Medical Genetics, Samsun University Faculty of Medicine, Samsun, Türkiye

**Keywords:** genetic diagnosis, MODY, monogenic diabetes, next-generation sequencing, pediatric diabetes, rare MODY subtypes

## Abstract

**Objective:**

Maturity-onset diabetes of the young (MODY) represents a genetically and clinically heterogeneous form of monogenic diabetes caused by defects in pancreatic β-cell function. Accurate molecular diagnosis is essential for distinguishing MODY from type 1 and type 2 diabetes, enabling precision-based management and targeted therapy. This study aimed to evaluate the genetic and clinical features of pediatric patients with MODY, to assess the prevalence of common and rare subtypes, and to report novel pathogenic variants identified in a Turkish cohort.

**Methods:**

This single-center, retrospective cohort study evaluated 81 pediatric patients with suspected MODY followed between 2022 and 2025. Genetic analysis was performed using targeted next-generation sequencing (NGS) panels, including *HNF4A*, *GCK*, *HNF1A*, *PDX1*, *HNF1B*, *NEUROD1*, *INS*, *ABCC8*, *KCNJ11*, *APPL1*, and *CEL*. Patients were selected based on the presence of at least two clinical features suggestive of MODY, as defined by the 2022 International Society for Pediatric and Adolescent Diabetes (ISPAD) Clinical Practice Consensus Guidelines. Demographic, biochemical, and clinical data were extracted from hospital records and analyzed descriptively.

**Results:**

Genetic variants were identified in 25 of 81 patients (30.9%), including pathogenic, likely pathogenic, and variants of uncertain significance (VUS). Of these, 22 variants were classified as pathogenic or likely pathogenic, corresponding to a diagnostic yield of 27.2%. The most frequently affected gene was *GCK* (72.0%), followed by *HNF1A* (8.0%), with single cases identified in *HNF1B*, *INS*, *PDX1*, *CEL*, *and KCNJ11*. Rare MODY subtypes collectively accounted for 20.0%. Three novel *GCK* variants c.1055T >C, c.1229A >C, and c.185_186insA were identified. One patient with syndromic features harbored a heterozygous 17q12 microdeletion encompassing *HNF1B*, approximately 1.5 Mb in size, and presented with global developmental delay, intellectual disability, epilepsy, dysmorphic facial features, persistent hypomagnesemia, and a bicornuate uterus with normal renal structure. Following genetic analysis, two patients had therapy adjustments based on the identified variants.

**Conclusion:**

This study underscores the clinical and genetic heterogeneity of MODY in the pediatric population and reinforces the value of comprehensive NGS panels for accurate diagnosis, even in patients who do not fully meet classical MODY criteria. The identification of novel *GCK* variants and the detection of rare subtypes further expand the mutational and phenotypic spectrum of pediatric monogenic diabetes. These findings highlight the importance of incorporating population-specific genomic data into clinical practice and of periodically re-evaluating gene–disease associations as new molecular and functional evidence emerges. Ultimately, the integration of molecular diagnostics into routine pediatric diabetes care will enhance diagnostic yield, optimize management, and improve long-term outcomes for affected families.

## 1. Introduction

Maturity-onset diabetes of the young (MODY) is a monogenic form of diabetes characterized by early-onset hyperglycemia, autosomal dominant inheritance, and pancreatic beta-cell dysfunction. MODY is distinct from both type 1 and type 2 diabetes based on its genetic, clinical, and metabolic features. Unlike type 1 diabetes, MODY is not associated with autoimmune markers and rarely requires insulin therapy [1, 2]. It also differs from type 2 diabetes by the general absence of obesity, insulin resistance, and dyslipidemia. These distinctions necessitate accurate diagnosis and individualized treatment strategies [3, 4].

To date, at least 14 genes have been historically described in association with MODY [5]. However, recent Clinical Genome Resource (ClinGen) and the Gene Curation Coalition evaluations have refined this list based on the strength of genetic and functional evidence. Currently, only a subset of genes, including *HNF1A*, *GCK*, *HNF4A*, *HNF1B*, *PDX1*, *NEUROD1*, *INS*, *ABCC8*, and *KCNJ11* are considered to have definitive or strong evidence supporting their causative role in MODY, while *APPL1* has only limited evidence. *CEL* currently has only moderate evidence, limited to specific frameshift variants in the VNTR region associated with combined endocrine and exocrine pancreatic dysfunction. In contrast, *KLF11*, *PAX4*, and *BLK* have been refuted as MODY-associated genes (https://search.clinicalgenome.org/kb/genes/). Consistently, several studies have supported these gene curation findings [6, 7].

Among the confirmed MODY genes, mutations in *HNF1A*, *GCK*, and *HNF4A* remain the most common, collectively accounting for approximately 90% of all genetically confirmed cases [3, 8]. *GCK*-MODY leads to stable and mild fasting hyperglycemia due to reduced glucokinase enzyme activity and typically does not require pharmacologic treatment, whereas *HNF1A*-MODY and *HNF4A*-MODY are associated with progressive β-cell dysfunction and are often highly responsive to sulfonylurea therapy [1, 9]. Less common but validated MODY subtypes such as those caused by variants in *HNF1B*, *PDX1*, *NEUROD1*, *INS*, *ABCC8*, *KCNJ11*, and *APPL1* are frequently associated with complex phenotypes, extrapancreatic manifestations, or clinical overlap with neonatal diabetes [10, 11]. Recognizing these rare forms is particularly important in pediatric and adolescent patients who do not fit traditional diabetes classifications.

Genetic analysis plays a pivotal role in the diagnosis of MODY by increasing diagnostic accuracy, guiding treatment decisions, predicting prognosis, and informing screening strategies for family member [8]. However, in the pediatric age group, monogenic diabetes is often diagnosed late. Many patients are initially misclassified as having type 1 diabetes and are started on insulin therapy without undergoing appropriate genetic evaluation [12]. Moreover, data regarding novel mutations and rare MODY subtypes remain limited in the literature.

In this study, we aimed to comprehensively evaluate the genetic and clinical characteristics of pediatric patients with MODY followed at a single center. The objectives were to define the clinical and phenotypic variability within this cohort, identify previously unreported (novel) mutations, and determine the prevalence of rare MODY subtypes. This study seeks to enhance awareness of MODY in the pediatric population and to support the integration of genetic diagnosis into routine clinical practice.

## 2. Materials and Methods

### 2.1. Study Design and Ethical Approval

This study was designed as a retrospective cohort study conducted at a single tertiary center. Ethical approval was obtained from the local ethics committee prior to data collection (Ethics Committee Decision No: GOKAEK 2024/5/2). Informed consent was obtained from the parents or legal guardians of all participants for both clinical evaluation and genetic testing, in accordance with the Declaration of Helsinki.

### 2.2. Study Population

A total of 81 pediatric patients followed by the pediatric endocrinology department between January 1, 2022, and January 1, 2025, were evaluated for suspected MODY based on clinical suspicion and features broadly consistent with the recommendations of the International Society for Pediatric and Adolescent Diabetes (ISPAD) 2022 Clinical Practice Consensus Guidelines. According to these guidelines, MODY should be considered in patients with early-onset diabetes (usually before 25 years of age), absence of obesity or marked insulin resistance, negative autoantibodies, preserved endogenous insulin secretion demonstrated by a detectable C-peptide level at least 3 years after diagnosis, a positive family history of diabetes in at least two consecutive generations, a mild and stable fasting hyperglycemia pattern consistent with a *GCK* phenotype, and absence of diabetic ketoacidosis (DKA) at onset. The ISPAD guidelines recommend performing genetic analysis in cases with a strong clinical suspicion of monogenic diabetes. In our study, we also reviewed previous pediatric studies and selected patients who met two or more of these ISPAD-recommended clinical features suggestive of MODY for genetic testing.

Among the 81 patients who underwent genetic testing, 25 were found to carry pathogenic, likely pathogenic, or variants of uncertain significance (VUS) in MODY-related genes and were included in the final analysis.

### 2.3. Clinical Data Collection

Demographic and clinical data were retrospectively extracted from patient files and electronic hospital records. Collected variables included: age, sex, age at diabetes diagnosis, presenting symptoms, presence of DKA at diagnosis, family history of diabetes, birth weight, comorbidities, anthropometric measurements (including body mass index [BMI] percentiles), and laboratory parameters at the time of diagnosis. These laboratory data comprised fasting blood glucose, HbA1c, basal C-peptide, fasting insulin, insulin autoantibodies (glutamic acid decarboxylase antibody [GADA], islet cell cytoplasmic antibody [ICA], insulin autoantibody [IAA]), insulin resistance, and lipid profile (total cholesterol, HDL cholesterol, LDL cholesterol, and triglycerides). The reference ranges applied during the study were as follows: fasting glucose: 74–106 mg/dL, HbA1c: 4.2%–6.0%, insulin: 2.6–24.9 µIU/mL, basal C-peptide: 1.1–4.4 ng/mL, HOMA-IR: <2.5, total cholesterol: 0–200 mg/dL, LDL cholesterol: 0–130 mg/dL, HDL cholesterol: 40–60 mg/dL, triglycerides: 0–200 mg/dL. GADA <17 IU/mL, IAA < 0.4 U/mL, and ICA < 1:4 were considered negative, based on the reference intervals used by the testing laboratory. GADA and IAA were measured using enzyme-linked immunosorbent assay (ELISA), whereas ICA was analyzed by indirect immunofluorescence assay (IFA). Zinc transporter 8 antibody (ZnT8A) and insulinoma-associated antigen-2 antibody (IA-2A) were not tested due to laboratory-related limitations during the study period.

In addition, the type of initial diabetes treatment, any treatment modifications following genetic diagnosis, and genetic test results were recorded.

### 2.4. Genetic Analysis

Genomic DNA was extracted from peripheral blood leukocytes using standard procedures. Targeted sequencing of MODY-associated genes, in accordance with the latest ClinGen Gene Curation Expert Panel recommendations, with definitive, strong, and moderate evidence including *HNF4A*, *GCK*, *HNF1A*, *PDX1*, *HNF1B*, *NEUROD1*, *INS*, *ABCC8*, *KCNJ11*, and *APPL1* and *CEL* was performed using multiple library preparation and hybridization capture kits, including the xGen DNA Library Prep EZ Kit and IDT hybridization capture reagents, as well as the GeneTopia Library Prep Kit and GeneTopia Hybridization Capture Kit. Despite the use of different protocols, all samples underwent enrichment for the same gene panel. Sequencing was carried out on either the Illumina platform or the GeneMind SURFSeq 5000 High-throughput Sequencing platform, with an average coverage depth of 70×–100× and ≥90% of targeted regions covered at this depth.

The processes of raw FASTQ data quality control, trimming, alignment, BAM file processing, depth/coverage analysis, and variant calling were performed through a standardized bioinformatic workflow. Initially, comprehensive quality control of all raw reads was conducted using FastQC, evaluating parameters, such as base quality scores, GC content, Q20–Q30 values, duplication rates, and insert size distribution. Subsequently, Fastp was used for per-sample read quality assessment, and FastQ Screen was applied to detect potential contaminations.

Adapter removal and read trimming were performed with Cutadapt, followed by alignment to the GRCh37/hg19 human reference genome using the BWA-MEM algorithm. Alignment outputs were processed through GATK MarkDuplicatesSpark for duplicate marking and sorting. General alignment statistics were obtained with samtools flagstat, and read length as well as mapping quality (MAPQ) distributions were calculated.

Mean sequencing depth and coverage rates at various thresholds (1×, 10×, 20×, 30×, 50×, and 100×) were determined using samtools depth and bedcov. Gene- and exon-level coverage assessments were performed with bedtools intersect, awk, mosdepth, and in-house R scripts, and the average depth and coverage percentage per gene were summarized. Base quality recalibration was carried out using GATK BaseRecalibrator and ApplyBQSR. The recalibrated BAM files were then processed for variant calling with GATK HaplotypeCaller (GVCF mode), followed by joint genotyping using GenotypeGVCFs and variant separation into SNPs and indels via SelectVariants. Variant filtering was performed with GATK VariantFiltration, applying stringent quality thresholds to exclude low-confidence variants.

High-confidence variants passing all filters were subsequently annotated using the Ensembl Variant Effect Predictor (VEP) to determine their functional impact and biological relevance. Only variants previously reported as pathogenic in Franklin, VarSome, HGMD Public, or ClinVar, or those with a minor allele frequency (MAF) <1% in public databases (gnomAD, ExAC, 1000 Genomes Project, dbSNP) were retained for downstream evaluation. Variant interpretation focused on protein-coding exonic regions and ±10 bp flanking intronic sequences, excluding variants with a read depth <10×.

To assess variant significance, multiple in silico predictive algorithms were employed, including DANN, MutationTaster, FATHMM, MetaSVM, MetaLR, GERP, phyloP, SiPhy, LRT, SIFT, and PROVEAN. Final classification was performed according to the American College of Medical Genetics and Genomics (ACMG) guidelines.

In addition, in one patient presenting with diabetes accompanied by global developmental delay, epilepsy, and dysmorphic facial features, chromosomal microarray (CMA) analysis was conducted due to syndromic suspicion. CMA was conducted using the Affymetrix CytoScan Optima platform (315 K). DNA extracted from peripheral blood was hybridized to microarray chips and analyzed using ChAS 3.1 software. Copy number variants (CNVs) interpretation was performed according to the ACMG guidelines, utilizing data from DECIPHER, DGV, OMIM, PubMed, and in-house laboratory databases. In this patient, MODY diagnosis was established based on the detection of a pathogenic deletion encompassing the *HNF1B* gene.

Segregation analysis was recommended for all index cases with identified variants. However, in majority of families, segregation testing could not be completed due to parental unavailability, lack of consent, or logistical constraints. Therefore, segregation data were not systematically included in this study.

### 2.5. Statistical Analysis

Descriptive statistics were used to summarize the data. Continuous variables were expressed as mean ± standard deviation (SD), and categorical variables as frequencies and percentages.

## 3. Results

### 3.1. Diagnostic Yield

Among the 81 pediatric patients who underwent molecular genetic testing due to clinical suspicion of MODY, 25 (30.9%) had a variant in MODY-related genes. Of these, 22 variants were classified as pathogenic or likely pathogenic, corresponding to a diagnostic yield of 27.2%.

### 3.2. Phenotypic and Clinical Characteristics

The 25 patients with identified variants in MODY-related genes, 8 (32.0%) were female and 17 (68.0%) were male. The mean age was 12.5 years (range: 3–17 years), and the mean age at diabetes diagnosis 9.7 (range: 1–16 years). Clinical characteristics of the 25 patients are presented in Table 1.

The most common reason for referral was incidentally detected hyperglycemia during routine medical evaluation (*n* = 15, 60.0%). Other presenting features included polyuria and polydipsia (*n* = 5, 20.0%), polyuria (*n* = 2, 8.0%), obesity (*n* = 2, 8.0%), and weight loss (*n* = 1, 4.0%).

All patients had a positive family history of diabetes. Six patients (24.0%) had a classic three-generation autosomal dominant inheritance pattern. Seven patients (28.0%) had a maternal history of gestational diabetes, which persisted as overt diabetes postpartum in three of them. Two patients (8.0%) had a parent with diagnosed diabetes, two (8.0%) had a sibling with confirmed MODY, one (4.0%) had a sibling with neonatal diabetes, and nine patients (36.0%) had a family history of diabetes limited to grandparents, with no clinical evidence of diabetes in the intervening generation.

Macrosomia was documented in one patient; all others were appropriate for gestational age (AGA).

According to BMI percentiles, three patients (12.0%) were underweight, 16 (64.0%) had normal weight, one (4.0%) was overweight, and five (20.0%) were obese.

At the time of diagnosis, the mean fasting glucose level was 146.8 mg/dL (range: 107–332 mg/dL), mean HbA1c was 7.43% (range: 6.1%–14.7%), and mean basal C-peptide was 1.53 ng/mL (range: 0.66–3.44 ng/mL), and mean fasting insulin was 9.2 µIU/mL (range: 2.86–25). Seven patients had insulin resistance defined as HOMA-IR > 2.5. HOMA-IR was not calculated in six patients because insulin therapy had already been initiated. Diabetic autoantibodies were negative in all but one patient who tested positive for GADA. In this patient, the GADA level was 46 IU/mL (range: 0–17 IU/mL), the highest recorded HbA1c was 8.3%, and the highest fasting glucose level was 264 mg/dL. The mean lipid profile values were as follows: triglycerides 102 mg/dL (range: 41–163), total cholesterol 156 mg/dL (120–251), LDL cholesterol 101.3 mg/dL (51–163), and HDL cholesterol 54.2 mg/dL (38–83). Three patients (12.0%) had hypercholesterolemia.

Regarding initial treatment, 19 patients (76.0%) were managed with diet alone, four (16.0%) received insulin plus dietary management, and two (8.0%) received insulin, metformin, and diet. Following genetic diagnosis, treatment modifications were made in two patients: in one patient with a *GCK* mutation who was initially on insulin, insulin therapy was discontinued due to decreasing insulin requirements; in another patient with a *KCNJ11* mutation, sulfonylurea therapy was initiated based on the genetic finding.

### 3.3. Genetic Findings and Variant Spectrum

**Figure 1:**
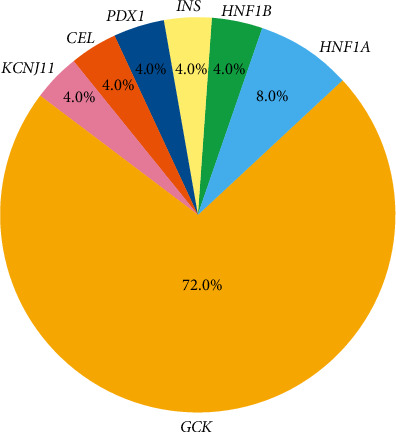
Distribution of genes associated with MODY. The pie chart illustrates the distribution of genes associated with MODY among 25 patients in the cohort. The most frequently affected gene was *GCK* (72%), followed by *HNF1A* (8%). Single cases were identified with mutations in *HNF1B*, *INS*, *PDX1*, *CEL*, and *KCNJ11* (each 4%).

Among the 25 patients, the most commonly affected gene was *GCK* (*n* = 18, 72.0%), followed by *HNF1A* (*n* = 2, 8.0%). Single cases were identified with mutations in *HNF1B*, *INS*, *PDX1*, *CEL*, *and KCNJ11* (each *n* = 1, 4.0%) (Figure 1). *GCK* and *HNF1A* accounted for 20 out of 25 patients (80.0%), whereas other subtypes were identified in the remaining five patients (20.0%). Genetic characteristics of the 25 patients are presented in Table 2.

A total of 23 unique variants were identified in 25 patients. Of these 23 distinct variants, 12 (52.2%) were classified as pathogenic, 8 (34.8%) as likely pathogenic, and 3 (13.0%) as VUS. The variants classified as of VUS were identified in the *GCK*, *PDX1*, and *INS* genes, and all had been previously reported in the literature. The three novel variants identified in this cohort—c.1055T >C (PM1, PP2, PM2, PP3, PM5, and PP5), c.1229A >C (PM1, PP2, PM2, PP3, and PM5), and c.185_186insA (PVS1 and PM2) were all located in the *GCK* gene, had not been previously reported in the literature, and were classified as likely pathogenic based on their respective ACMG criteria.

Among the 23 variants, 14 (60.9%) were missense, three (13.0%) nonsense, three (13.0%) frameshift, and two (8.7%) splice site mutations. In addition, one patient had a heterozygous deletion at chromosome 17q12, approximately 1.5 Mb in size, consistent with the 17q12 microdeletion syndrome region, in which exons 1–9 of the *HNF1B* gene were deleted. In this patient, global developmental delay, intellectual disability, epilepsy, and dysmorphic features were present. The patient also showed persistent hypomagnesemia, with serum magnesium levels ranging between 0.83 and 1.23 mg/dL (normal range: 1.8–2.6 mg/dL). Renal function tests were within normal limits. Liver enzymes were mildly elevated (ALT: 69 U/L [0–50], AST: 45 U/L [0–50]). Genitourinary ultrasonography revealed a bicornuate uterus without renal cysts or other structural renal abnormalities.

### 3.4. Clinical and Biochemical Characteristics of *GCK*-MODY

In the cohort, 18 patients (72.0%) carried a *GCK* mutation. Two of these patients were siblings. The mean age at genetic diagnosis was 11.2 years, and the mean age at diabetes diagnosis was 8.6 years. Hyperglycemia was most frequently detected incidentally during routine laboratory testing (*n* = 12, 66.7%). Most patients were asymptomatic at the time of diagnosis, and none presented with DKA. None of the patients were classified as obese. All six patients with a maternal history of gestational diabetes were identified as having a *GCK* mutation. At the time of diagnosis, the mean fasting glucose level was 125 mg/dL, the mean HbA1c was 6.7%, and the mean basal C-peptide was 1.4 ng/mL. Diabetes-related autoantibodies were negative in all individuals, apart from a single patient who tested positive for GADA. None of the *GCK*-MODY patients had dyslipidemia, and five patients had insulin resistance defined as HOMA-IR >2.5. Initial treatment at diagnosis consisted of diet alone in 17 out of 18 patients (94.4%). One patient (5.6%) received insulin as the initial therapy owing to the detection of GADA positivity. Over time, insulin therapy was discontinued due to decreasing insulin requirements.

## 4. Discussion

The clinical and genetic characterization of monogenic diabetes in childhood remains a major diagnostic challenge, particularly in patients who do not conform to the classical MODY phenotype. Although MODY is typically associated with nonobese individuals, preserved C-peptide levels, and a positive multigenerational family history, pediatric presentations often deviate from this pattern. Some children may present with obesity, mild insulin resistance, or even ketoacidosis, making the clinical distinction from type 1 or type 2 diabetes difficult [3, 13].

In this context, our single-center cohort provides insight into the real-world diagnostic complexity of MODY in the pediatric population. Among 81 children tested for suspected MODY, 22 (27.2%) were found to carry clinically relevant variants in MODY-related genes, yielding a diagnostic rate comparable to previous pediatric studies (20%–50%) [14–16]. This result demonstrates that genetic confirmation is achievable even in clinically heterogeneous populations when appropriate selection criteria are applied.

To optimize diagnostic efficiency, patients in our study were selected based on the presence of at least two clinical features suggestive of MODY, consistent with previous pediatric cohort strategies [15, 17, 18]. While the 2022 ISPAD Consensus Guidelines emphasize clinical suspicion as the key determinant, they do not specify a minimum number of required features. To ensure both diagnostic efficiency and clinical applicability, we adopted a practical approach consistent with prior literature, which has similarly used the “two or more” threshold to guide molecular testing in suspected MODY cases. This method provides a balanced strategy between diagnostic yield and resource efficiency, ensuring that potential MODY cases are not overlooked while minimizing unnecessary testing in children with low pretest probability. Overall, these findings reinforce the need to consider MODY even in atypical or overlapping clinical presentations and highlight the value of integrating molecular diagnostics into the routine evaluation of pediatric diabetes.

The clinical heterogeneity observed in pediatric MODY extends beyond biochemical features and is also evident in demographic characteristics, such as age at onset. In our cohort, the mean age at diabetes diagnosis was 9 years and 11 months, which aligns with previous pediatric studies reporting onset typically between 8 and 12 years [4, 15, 19]. Although MODY can be diagnosed at any point from infancy to early adulthood, earlier onset in childhood often reflects increased clinical awareness and access to genetic testing, whereas delayed recognition may occur in cases with mild or asymptomatic hyperglycemia [20].

In our cohort, males accounted for approximately two-thirds of all genetically confirmed cases, indicating a modest male predominance. Although MODY is classically described as having no consistent sex predilection, several pediatric cohorts have reported a similar trend toward male overrepresentation [21, 22]. The underlying cause of this imbalance remains unclear but is likely multifactorial, involving a combination of genetic, hormonal, and methodological factors. From a biological standpoint, sex-related differences in hepatic glucose metabolism and insulin sensitivity during puberty may transiently accentuate hyperglycemia in boys, thereby increasing the likelihood of clinical recognition. In addition, X-linked or sex-influenced modifier genes that modulate β-cell function or insulin responsiveness have been hypothesized to contribute to subtle phenotypic variability, though definitive evidence is lacking. Sociocultural and methodological factors may further influence this distribution. Boys are often more likely to undergo routine metabolic or sports-related screening, which may facilitate earlier detection of mild, asymptomatic hyperglycemia. The predominance observed in this cohort could, therefore, reflect referral or detection bias, particularly within a tertiary care setting where diagnostic testing is guided by presentation patterns and clinician awareness.

Several patients in our cohort exhibited clinical features that deviated from the conventional MODY phenotype, including obesity, mild insulin resistance, and isolated autoantibody positivity. Although obesity is classically considered atypical for MODY, recent pediatric cohorts have reported that genetically confirmed cases may present with elevated BMI values [22, 23]. This trend likely reflects the increasing prevalence of obesity in the general population and the phenotypic overlap with type 2 diabetes, which may obscure recognition of an underlying monogenic etiology.

Insulin resistance (HOMA-IR > 2.5) was detected in seven patients. While MODY primarily results from pancreatic β-cell dysfunction, mild or transient insulin resistance can coexist, particularly during puberty or in individuals with excess adiposity. Previous studies have demonstrated that insulin resistance may also occur in certain rare MODY subtypes and specific populations, potentially as a result of hormonal influences, genetic modifiers, or environmental factors [24–26].

Autoantibody screening revealed GADA positivity in one patient who was later confirmed to have *GCK*-MODY. In patients with MODY, autoantibody positivity is usually below 1% and most often involves a single antibody detected at a low titer [3, 27]. In large cohort studies, positivity for a single autoantibody has been reported in approximately 11.4% of MODY patients, whereas multiple autoantibody positivity is exceedingly rare. The presence of autoantibodies does not exclude a diagnosis of MODY, as single and low-titer antibodies are not highly specific for type 1 diabetes mellitus [14, 28]. Therefore, in children with a clinical suspicion of MODY who present with isolated autoantibody positivity, molecular confirmation is warranted after careful evaluation of family history and phenotypic features.

Interestingly, patients who were positive for GADA, had low insulin requirements, and preserved C-peptide levels despite poor glycemic control, suggesting a transient phase of endogenous insulin secretion compatible with the honeymoon period of type 1 diabetes. Given the detection of a heterozygous *GCK* variant, the possibility of a dual diagnosis of type 1 diabetes and *GCK*-MODY should be considered, as highlighted in recent reports [29, 30].

Taken together, these findings demonstrate that pediatric MODY may manifest with atypical features—such as overweight, mild insulin resistance, or isolated autoantibody positivity—highlighting the importance of a comprehensive, genetics-based diagnostic approach rather than rigid adherence to classical phenotypic definitions [3, 14].

Interestingly, six of the mothers of patients with *GCK*-MODY in our cohort had been diagnosed with gestational diabetes during pregnancy. These cases suggest that some women diagnosed with gestational diabetes may, in fact, have *GCK*-MODY, as heterozygous *GCK* mutations cause mild, lifelong fasting hyperglycemia that is present from birth but often first recognized during pregnancy, when routine glucose testing is performed [31]. In various studies, *GCK*-MODY has been identified in approximately 1.5%–3.6% of women diagnosed with gestational diabetes [32]. Distinguishing *GCK*-MODY from gestational diabetes is crucial, as it has significant implications for clinical management, treatment decisions during pregnancy, and genetic counseling for both the patient and her family.

Genetic testing serves as a cornerstone of precision medicine by enabling accurate diagnosis and guiding individualized treatment strategies tailored to the underlying molecular defect. In our cohort, molecular confirmation directly influenced therapeutic decisions: insulin therapy was successfully discontinued in a patient with a *GCK* mutation after reclassification of the diabetes type, and sulfonylurea therapy was initiated in a patient with a *KCNJ11* mutation based on the genetic finding. These examples underscore how the integration of molecular diagnostics into clinical practice can optimize management, prevent unnecessary insulin use, and improve long-term outcomes in pediatric patients with monogenic diabetes [33, 34].

The most frequently identified subtype in this cohort was *GCK*-MODY (72%), followed by *HNF1A*-MODY (8%). This distribution aligns with global trends but also reflects regional differences in MODY subtype prevalence. Beyond these common forms, rare MODY subtypes accounted for 20% of cases in our cohort, which is higher than the <5% typically reported in the literature [35]. It should also be noted that our gene selection strategy was based on the latest ClinGen Gene Curation Expert Panel recommendations, which exclude genes with limited or refuted evidence for MODY association such as *BLK*, *KLF11*, and *PAX4* from diagnostic panels (https://search.clinicalgenome.org/kb/genes/). This methodological refinement may partly account for differences in gene distribution between our study and earlier reports that included such genes in their analyses.

In Europe, the United States, and many other countries, *GCK*-MODY and *HNF1A*-MODY are the two most common subtypes, together accounting for approximately 60%–90% of all MODY cases [36]. In countries, such as Portugal, Hungary, and Poland, *GCK*-MODY has been reported as the most frequent subtype [36, 37]. In contrast, in some Western European countries such as the United Kingdom and Norway, *HNF1A*-MODY predominates (e.g., in the UK, *HNF1A*-MODY 63% and *GCK*-MODY 32%) [37]. In Europe, *HNF1B*-MODY and *INS*-MODY together represent approximately 2%–3% of genetically confirmed cases, while *PDX1* and *CEL* variants occur in fewer than 1% [15, 36].

In Asian populations, the distribution of MODY subtypes shows greater heterogeneity compared with European cohorts. *HNF4A* mutations are markedly less common (2%–5%) [14, 38]. Rare subtypes are also more frequent in Asia, with *HNF1B* and *ABCC8* variants representing up to 5%–10% of cases in Japan and China, and *KLF11* and *NEUROD1* mutations reported in upto 4% of Chinese cohorts [14, 38, 39]. Reports from India have described an even broader mutation spectrum, including *ABCC8*, *HNF1B*, and *INS* variants, collectively accounting for a substantial proportion of monogenic diabetes diagnoses [40, 41].

The Turkish cohort demonstrated a pattern partially consistent with European reports, showing a higher prevalence of the *GCK* subtype and comparatively lower frequencies of *HNF1A* and *HNF4A* mutations [9]. In a multicenter study, including 169 pediatric patients with monogenic diabetes, *GCK*-MODY was identified as the most common subtype, accounting for 59.2% of cases, whereas *HNF1A*-MODY and *HNF4A*-MODY were detected in 18.3% and 1.2% of patients, respectively [42]. In another national study, *GCK* mutations were found in 18 of 43 patients (42%) and *HNF1A* mutations in four patients (9%), while an *HNF4A* mutation was detected in only one patient [43].

Indeed, some published studies from Türkiye have reported compatible findings. In certain cohorts, the proportion of rare variants has been shown to reach 10%–30% [19, 44]. In the study by Aydogan et al. [45], no pathogenic mutation was identified in the *GCK* gene; instead, 17 different variants were detected in 15 of 51 adult patients (29.4%) across the *PDX1*, *HNF1B*, *KLF11*, *CEL*, *BLK*, *and ABCC8* genes. Kanca Demirci et al. [46] reported *GCK* mutations in only two of 22 pediatric MODY cases. In another study by Haliloğlu et al. [47], which focused exclusively on the *GCK* gene, 54 patients were analyzed, and pathogenic, likely pathogenic, or intronic VUS were detected in 14 individuals [47].

Taken together, these findings demonstrate that the distribution of MODY subtypes may vary geographically, reflecting differences in genetic background and ethnic composition. Variability in study design, patient selection criteria, and the clinical thresholds used to suspect monogenic diabetes may further contribute to these discrepancies among populations. In addition, differences in access to molecular diagnostics and in the use of targeted versus comprehensive next-generation sequencing (NGS) panels can influence the detection rates of both common and rare subtypes. This highlights the importance of employing broad and standardized genetic testing strategies, along with maintaining population-specific variant databases, to improve diagnostic yield and refine genotype–phenotype correlations in monogenic diabetes.

The patient with *HNF1B*-MODY in our cohort exhibited a syndromic form of the disease characterized by global developmental delay, intellectual disability, epilepsy, dysmorphic features, persistent hypomagnesemia, mildly elevated liver enzymes, and a bicornuate uterus. CMA analysis identified a 1.5 Mb heterozygous deletion at chromosome 17q12 encompassing exons 1–9 of the *HNF1B* gene, consistent with the 17q12 microdeletion syndrome region. This recurrent microdeletion is associated with a highly variable multisystem phenotype, most commonly, including renal malformations, early-onset diabetes, and neurodevelopmental abnormalities [48–50]. Interestingly, our patient displayed the neurodevelopmental and metabolic manifestations typical of *HNF1B* haploinsufficiency but lacked renal involvement, highlighting the phenotypic heterogeneity of 17q12 deletion syndrome. This case underscores the diagnostic value of CMA as a first-tier test for patients with diabetes accompanied by neurodevelopmental delay and dysmorphic or multisystemic features, as single-gene sequencing approaches may fail to detect pathogenic CNVs.

Among the *HNF1A*-MODY patients, both were obese at the time of diagnosis. One patient had a history of macrosomia and hypercholesterolemia, while the other presented with insulin resistance requiring insulin and metformin therapy. These features—macrosomia, dyslipidemia, and insulin resistance—are more commonly associated with type 2 diabetes and may obscure the clinical suspicion of monogenic diabetes in pediatric patients. Although obesity and insulin resistance are not typical findings in *HNF1A*-MODY, emerging evidence suggests that environmental and metabolic modifiers, such as maternal hyperglycemia during pregnancy or underlying metabolic syndrome, may influence disease expression [51, 52]. Macrosomia, in particular, is more frequently associated with *HNF4A*-MODY due to its impact on fetal insulin secretion, but has also been occasionally reported in *HNF1A*-MODY, potentially reflecting unrecognized maternal hyperglycemia [53]. These observations emphasize the importance of considering MODY in pediatric patients who do not fully meet classical criteria, and they support the use of genetic testing to clarify diagnosis in cases with overlapping phenotypic features.

A notable case in our cohort involved a *KCNJ11* mutation identified in a patient presenting with early-onset, non-autoimmune diabetes. Segregation analysis revealed the same variant in the patient's mother—who had a history of gestational diabetes—and in a sibling previously diagnosed with neonatal diabetes. *KCNJ11* mutations can present with a broad clinical spectrum, including transient or permanent neonatal diabetes, MODY-like diabetes in childhood or early adulthood, type 2 diabetes phenotypes, and congenital hyperinsulinism. Even within the same family, individuals carrying the same pathogenic *KCNJ11* mutation may exhibit significant variability in age of onset and disease severity; while some develop diabetes in the neonatal period, others may present later in life with a milder, MODY-like phenotype [54–56]. This intrafamilial heterogeneity underscores the variable expressivity and incomplete penetrance of KCNJ11-related diabetes, highlighting the need for individualized clinical evaluation and long-term follow-up. The efficacy of sulfonylureas in patients with KCNJ11-related diabetes has been well established, with studies demonstrating successful transition from insulin to oral therapy in the majority of cases [57]. In our cohort, a patient with a *KCNJ11* mutation was transitioned to sulfonylurea therapy following molecular diagnosis, resulting in improved glycemic control and illustrating the clinical value of personalized, gene-informed treatment strategies.

Three novel *GCK* variants (c.1055T > C, c.1229A > C, and c.185_186insA) were identified and classified as likely pathogenic based on ACMG criteria. These variants were absent from population databases and affected highly conserved residues or introduced frameshifts leading to early truncation, all of which support their pathogenic potential. The discovery of novel *GCK* mutations represents a major strength of this study, as this gene exhibits one of the most heterogeneous mutational spectra among all MODY-related genes, with more than 600 distinct variants described worldwide [32, 38]. By adding three previously undescribed variants, our findings expand the existing *GCK* variant spectrum and contribute to the refinement of genotype–phenotype correlations in monogenic diabetes. These results provide a valuable foundation for future functional and structural studies aimed at elucidating glucokinase activity and enhancing clinical variant interpretation in pediatric populations.

The *CEL* frameshift variant identified in our cohort c.1974del (p.Val659CysfsTer45) was classified as pathogenic according to ACMG criteria. This variant, located in exon 11, results in a premature stop codon and is predicted to cause loss of normal protein function through nonsense-mediated decay. The *CEL* gene encodes carboxyl ester lipase, and although it has been proposed as a candidate MODY gene, the current ClinGen Gene Curation Expert Panel assigns it moderate evidence for disease association (https://search.clinicalgenome.org/kb/genes/). Single-base deletions within the VNTR-containing C-terminal region cause a frameshift in the *CEL* protein, leading to the formation of an abnormal C-terminal tail. These mutant proteins accumulate within the cell, form protein aggregates, and induce endoplasmic reticulum stress. As a result, both exocrine and endocrine pancreatic dysfunction develop. However, the clinical significance of other *CEL* variants remains limited and controversial. [58–60]. The c.1974del variant maps to exon 11 within this VNTR-containing region, consistent with the canonical hotspot for *CEL*-MODY-associated single-base deletions. Although our patient did not exhibit overt exocrine pancreatic dysfunction, early-onset non-autoimmune diabetes with preserved C-peptide levels suggested a mild endocrine-predominant phenotype. Given its predicted protein-truncating nature, absence in population databases, and phenotypic concordance with previously reported *CEL*-MODY cases, this variant remains a plausible contributor to disease. Nevertheless, additional segregation and functional studies are needed to further elucidate the pathogenic role of *CEL* variants and refine their clinical interpretation within the MODY spectrum.

In our cohort, 13 variants were classified as pathogenic, 10 as likely pathogenic, and three as VUS. The three VUS were identified in the *GCK*, *INS*, and *PDX1* genes, all of which have been previously reported in the literature and are located in regions functionally relevant to beta-cell development or insulin secretion. Although they were categorized as VUS according to ACMG guidelines due to limited population frequency data, lack of functional validation, or insufficient segregation evidence, their recurrence in monogenic diabetes databases and the phenotypic consistency observed in our patients support a potential causal role. In each of these cases, the clinical presentation—including early-onset non-autoimmune diabetes and preserved C-peptide levels—strengthened the suspicion that these variants may indeed be disease-causing. As previously noted in the literature, some variants initially classified as VUS have been reclassified over time as new clinical and functional data become available [61]. Therefore, continued clinical follow-up and periodic reevaluation of VUS are essential in pediatric MODY cohorts where phenotype–genotype correlation directly informs diagnosis and management.

The use of a comprehensive NGS panel that included all genes with definitive, strong, or moderate evidence for MODY substantially improved the diagnostic yield in our cohort. This broad approach enabled the identification of both common subtypes and rare or atypical forms, such as *CEL*, *PDX1*, and *KCNJ11*, which might have been missed using phenotype-driven or gene-limited testing strategies. In addition, three novel *GCK* variants were identified, underscoring that even in well-characterized genes, novel or previously unreported alterations continue to emerge. NGS-based gene panels provide a rapid, comprehensive, and cost-effective diagnostic approach for children with non-autoimmune and atypical forms of diabetes, enabling personalized management and follow-up strategies. Therefore, they are recommended as a primary diagnostic tool in this patient group [62]. However, interpretation challenges remain, particularly when variants are classified as VUS. This highlights the ongoing need to integrate clinical phenotyping with molecular data and to periodically re-evaluate ambiguous findings as new evidence becomes available.

The strengths of this study include its focus on a well-characterized and homogeneous pediatric population, the identification of previously unreported genetic mutations, and the detection of rare MODY subtypes within a systematically evaluated cohort.

This study has several limitations that should be considered when interpreting the findings. Being conducted at a single tertiary center may limit the generalizability of the results. The relatively small cohort size could reduce the ability to detect rare genotypic or phenotypic associations. The study included a selected group of patients with clinical suspicion of MODY, which may limit the generalizability of the findings to the broader pediatric diabetes population. As this was a retrospective study, data availability and consistency depended on existing medical records, which may have resulted in incomplete documentation for certain variables. Consequently, some clinical and biochemical data were missing for a few patients; for example, the full spectrum of type 1 diabetes antibodies could not be tested in all cases, including the ZnT8A and IA-2A protein 2. The exact age at diabetes onset among affected family members, particularly parents, was not consistently available in patient records, and therefore, was not included in the analysis. This limitation may have restricted the assessment of familial disease patterns.

Further studies are warranted to functionally characterize the novel variants identified in this study and to confirm their pathogenicity through experimental validation. Multicenter investigations involving larger and more diverse pediatric cohorts will be important to enhance the generalizability of these findings and to better define the genotype–phenotype spectrum of MODY in children. As ClinGen expert panels continue to refine gene–disease validity and variant interpretation frameworks, future re-evaluation of certain variants may provide new insights into their clinical significance as additional evidence becomes available.

## 5. Conclusion

This study underscores the clinical and genetic heterogeneity of MODY in the pediatric population and highlights the importance of comprehensive genetic testing in patients with nonautoimmune diabetes. The identification of both common and rare subtypes—including novel variants—demonstrates the diagnostic power of NGS-based panels. Our findings support early genetic testing regardless of strict adherence to classical MODY criteria and emphasize the need for integrating molecular diagnostics into routine pediatric diabetes evaluation. Broader access to genetic testing and periodic reclassification of ambiguous variants will be essential to optimize diagnosis, management, and family-based care in monogenic diabetes. Moreover, incorporating CNV analysis into MODY testing protocols may further enhance diagnostic yield, particularly in syndromic cases where structural abnormalities may coexist with monogenic diabetes.

## Figures and Tables

**Table 1:** Clinical characteristics of MODY patients.

Patient ID	Sex	Age (years)	Age at DM diagnosis (years)	Presenting symptoms	Birth weight	BMI (percentile)	Family history of DM: affected relatives	Fasting blood glucose (mg/dL)	HbA1c (%)	Basal C, peptide (ng/mL)	Insulin (μIU/mL)	IR (HOMA-IR)	Diabetes autoantibodies	TG (mg/dL)	TC (mg/dL)	LDL-C (mg/dL)	HDL-C (mg/dL)	Initial treatment
P1	M	17	13	Polydipsia, polyuria	AGA	99.51	Maternal grandmother, maternal aunt	145	6.3	1.7	24.1	8.63	Negative	137	246	163	56	Diet
P2	M	3	1	Polyuria	AGA	78.23	Mother, father, paternal grandfather	120	6.8	1.3	4.9	1.45	Negative	66	136	83	40	Diet
P3	M	12	6	Asymptomatic hyperglycemia	AGA	48.01	Mother (gestational DM progressing to overt DM), maternal grandfather	111	6.21	1.72	13.3	3.64	Negative	41	180	113	59	Diet
P4	F	11	7	Polydipsia, polyuria	AGA	49.2	Maternal grandmother	120	6.4	0.66	2.86	0.85	Negative	59	191	92	64	Diet
P5	M	6	5	Asymptomatic hyperglycemia	AGA	50	Mother (gestational DM), paternal grandmother	122	6.1	3.44	4.9	1.48	Negative	67	183	100	70	Diet
P6	M	12	9	Asymptomatic hyperglycemia	AGA	68.44	Mother (gestational DM progressing to overt DM), maternal grandfather	116.2	6.17	1.41	5.8	1.66	Negative	163	169	83	53	Diet
P7	M	11	8	Asymptomatic hyperglycemia	AGA	93.82	Father, paternal grandmother	125	6.8	1.06	6.9	2.13	Negative	135	172	105	40	Diet
P8	F	4	4	Polydipsia, polyuria	AGA	44.04	Mother (gestational DM progressing to overt DM)	112.4	6.4	1.08	4.55	1.26	Negative	91	173	104	51	Diet
P9	M	10	6	Asymptomatic hyperglycemia	AGA	67	Mother (gestational DM progressing to overt DM)	122	6.48	1.26	7.78	2.34	Negative	79	164	89	59	Diet
P10	M	15	14	Polydipsia, polyuria	AGA	2.07	Mother (gestational DM)	135	7.75	1.16	4.89	1.63	Negative	110	172	91	59	Diet
P11	M	5	4	Polyuria	AGA	12.92	Maternal grandmother	109	6.2	0.83	3.9	1.05	Negative	147	157	91	47	Diet
P12	F	16	13	Asymptomatic hyperglycemia	AGA	22.06	Father, paternal aunt, paternal grandfather	140	6.22	1.16	9.8	3.4	Negative	99	175	94	61	Diet
P13	M	17	10	Asymptomatic hyperglycemia	AGA	17.88	Brother (*GCK*-MODY)	107	6.69	0.86	4.2	1.1	Negative	126	173	98	50	Diet
P14	M	17	13	Asymptomatic hyperglycemia	AGA	3.9	Brother (*GCK*-MODY)	120	6.89	1.94	9.37	2.78	Negative	86	143	87	39	Diet
P15	M	16	15	Asymptomatic hyperglycemia	AGA	29.46	Mother	116	6.9	2.64	15.3	4.38	Negative	138	120	51	41	Diet
P16	M	11	10	Weight loss	AGA	1.5	Father, paternal grandfather	205.6	8.3	0.95	4.2	NA^a^	GADA positive	72	161	104	46	Insulin, diet
P17	M	14	12	Asymptomatic hyperglycemia	AGA	64.06	Maternal grandmother	116	6.64	1.21	17.3	4.95	Negative	49	193	100	83	Diet
P18	M	6	4	Asymptomatic hyperglycemia	AGA	45.62	Maternal grandmother	115.8	6.91	1.27	4.68	1.34	Negative	89	196	114	67	Diet
P19	F	16	14	Asymptomatic hyperglycemia	AGA	14.46	Maternal grandmother	138	6.9	1.16	5.2	1.77	Negative	112	156	96	38	Diet
P20	F	17	16	Obesity	AGA	99.73	Mother, father	332	11.5	1.26	8.86	NA^a^	Negative	131	162	91	41	Insulin, metformin, diet
P21	F	16	14	Asymptomatic hyperglycemia	LGA	>99.98	Father, paternal uncle, paternal aunt, paternal grandfather	261	10.98	2.73	7.37	NA^a^	Negative	91	214	134	62	Insulin, diet
P22	F	17	13	Asymptomatic hyperglycemia	AGA	15.15	Paternal grandmother	231	14.7	2.08	3.7	NA^a^	Negative	153	182	94	53	Insulin, diet
P23	M	15	13	Obesity	AGA	99.95	Paternal grandmother, maternal grandmother	111	6.5	1.24	20.5	5.61	Negative	96	182	102	61	Diet
P24	F	16	8	Polydipsia, polyuria	AGA	96.93	Mother (gestational DM), brother (neonatal DM)	146.3	8.14	1.46	11	NA^a^	Negative	152	251	153	68	Insulin, diet
P25	M	13	12	Asymptomatic hyperglycemia	AGA	42.07	Paternal grandfather	195	6.9	2.8	25	NA^a^	Negative	61	160	101	47	Insulin, metformin, diet

Abbreviations: AGA, appropriate for gestational age; BMI, body mass index; DM, diabetes mellitus; F, female; GADA, glutamic acid decarboxylase antibody; HDL-C, high-density lipoprotein cholesterol; IR, insulin resistance; LDL-C, low-density lipoprotein cholesterol; LGA, large for gestational age; M, male; NA, not applicable; TC, total cholesterol; TG, triglyceride.

^a^HOMA-IR was not calculated in patients receiving exogenous insulin therapy.

**Table 2:** Genetic characteristics of MODY patients.

Patient ID	MODY Type	Gene	Genetic result	Zygosity	Mutation type	Mutation location	Pathogenicity classification of variants	ACMG evidence	Known/novel
P1	*CEL*-MODY	*CEL*	*CEL* (NM_001807.6):c.1974del p.(Val659CysfsTer45)	Heterozygous	Frameshift	Exon 11	LP	PVS1, PM2, BP6	Known
P2	*GCK*-MODY	*GCK*	*GCK* (NM_000162.5):c.1178T >C p.(Met393Thr)	Heterozygous	Missense	Exon 9	P	PS4, PM1, PP2, PM2, PP3, PP5	Known
P3	*GCK*-MODY	*GCK*	*GCK* (NM_000162.5):c.185_186insA p.(Arg63AlafsTer21)	Heterozygous	Frameshift	Exon 2	LP	PVS1, PM2	Novel
P4	*GCK*-MODY	*GCK*	*GCK* (NM_000162.5):c.802G >A p.(Glu268Lys)	Heterozygous	Missense	Exon 7	P	PP1, PP4, PS4, PM1, PP2, PM2, PP3, PP5	Known
P5	*GCK*-MODY	*GCK*	*GCK* (NM_000162.5):c.1055T >C p.(Leu352Pro)	Heterozygous	Missense	Exon 9	LP	PM1, PP2, PM2, PM5, PP3, PP5	Novel
P6	*GCK*-MODY	*GCK*	*GCK* (NM_000162.5):c.1155dup p.(Leu386AlafsTer73)	Heterozygous	Frameshift	Exon 9	LP	PVS1, PM2, PP5	Known
P7	*GCK*-MODY	*GCK*	*GCK* (NM_000162.5):c.1253+8C >A	Heterozygous	Splice-site	Intron 9	VUS	PM2, BP4	Known
P8	*GCK*-MODY	*GCK*	*GCK* (NM_000162.5):c.1318G >T p.(Glu440Ter)	Heterozygous	Nonsense	Exon 10	P	PVS1, PP1, PP4, PS4, PM2, PP5	Known
P9	*GCK*-MODY	*GCK*	*GCK* (NM_000162.5):c.556C >T p.(Arg186Ter)	Heterozygous	Nonsense	Exon 5	P	PVS1, PM2, PS4, PP5	Known
P10	*GCK*-MODY	*GCK*	*GCK* (NM_000162.5):c.617C >T p.(Thr206Met)	Heterozygous	Missense	Exon 6	P	PS4, PP3, PM2, PM5, PM1, PP1, PP2, PS3, PP5	Known
P11	*GCK*-MODY	*GCK*	*GCK* (NM_000162.5):c.667G >A p.(Gly223Ser)	Heterozygous	Missense	Exon 6	P	PM3, PP3, PM2, PM5, PM1, PP2, PS3, PP1, PP5	Known
P12	*GCK*-MODY	*GCK*	*GCK* (NM_000162.5):c.667G >A p.(Gly223Ser)	Heterozygous	Missense	Exon 6	P	PM3, PP3, PM2, PM5, PM1, PP2, PS3, PP1, PP5	Known
P13	*GCK*-MODY	*GCK*	*GCK* (NM_000162.5):c.683C >T p.(Thr228Met)	Heterozygous	Missense	Exon 7	P	PS2, PP1, PP3, PM2, PM5, PM1, PP4, PS4, PP2, PS3, PM3, PP5	Known
P14	*GCK*-MODY	*GCK*	*GCK* (NM_000162.5):c.683C >T p.(Thr228Met)	Heterozygous	Missense	Exon 7	P	PS2, PP1, PP3, PM2, PM5, PM1, PP4, PS4, PP2, PS3, PM3, PP5	Known
P15	*GCK*-MODY	*GCK*	*GCK* (NM_000162.5):c.751A >G p.(Met251Val)	Heterozygous	Missense	Exon 7	P	PS4, PM2, PM5, PM1, PP3, PP2, PP1, PP5	Known
P16	*GCK*-MODY	*GCK*	*GCK* (NM_000162.5):c.950A >C p.(His317Pro)	Heterozygous	Missense	Exon 8	LP	PM2, PM5, PM1, PP2	Known
P17	*GCK*-MODY	*GCK*	*GCK* (NM_033507.3):c.1229A >C p.(Asp410Ala)	Heterozygous	Missense	Exon 9	LP	PP3, PM2, PM1, PM5, PP2	Novel
P18	*GCK*-MODY	*GCK*	*GCK* (NM_033507.3):c.509A >G p.(Lys170Arg)	Heterozygous	Missense	Exon 5	LP	PP3, PM2, PM1, PP2, PP5	Known
P19	*GCK*-MODY	*GCK*	*GCK* (NM_033507.3):c.547G >A p.(Val183Met)	Heterozygous	Missense	Exon 5	P	PS4, PP3, PM2, PM5, PM1, PP2, PS3, PP1, PP5	Known
P20	*HNF1A*-MODY	*HNF1A*	*HNF1A* (NM_000545.8):c.326+1G >A	Heterozygous	Splice-site	Intron 1	P	PVS1, PP1, PM2, PS1, PP4, PP5	Known
P21	*HNF1A*-MODY	*HNF1A*	*HNF1A* (NM_000545.8):c.970C >T p.(Gln324Ter)	Heterozygous	Nonsense	Exon 5	LP	PVS1, PM2	Known
P22	*HNF1B*-MODY	*HNF1B*	arr[GRCh37] 17q12(34831962–36244358)x1	Heterozygous	Deletion	Exon 1–9	P	1 (1A, 2A–2E, 2H, 3A, 4L–4O)	Known
P23	*INS*-MODY	*INS*	*INS* (NM_000207.3):c.202C >A p.(Leu68Met)	Heterozygous	Missense	Exon 3	VUS	PM2, PP2	Known
P24	*KCNJ11*-MODY	*KCNJ11*	*KCNJ11* (NM_000525.4):c.685G >A p.(Glu229Lys)	Heterozygous	Missense	Exon 1	P	PS4, PP3, PM2, PM1, PP2, PP5	Known
P25	*PDX1*-MODY	*PDX1*	*PDX1* (NM_000209.4):c.467C >T p.(Ala156Val)	Heterozygous	Missense	Exon 2	VUS	PM2, PM1	Known

Abbreviations: Het, heterozygous; LP, likely pathogenic; P, pathogenic; VUS, variant of uncertain significance.

## Data Availability

The data that support the findings of this study are available on request from the corresponding author. The data are not publicly available due to privacy or ethical restrictions.
